# Hyperbaric Oxygen Therapy with Iloprost Improves Digit Salvage in Severe Frostbite Compared to Iloprost Alone

**DOI:** 10.3390/medicina57111284

**Published:** 2021-11-22

**Authors:** Marie-Anne Magnan, Angèle Gayet-Ageron, Pierre Louge, Frederic Champly, Thierry Joffre, Christian Lovis, Rodrigue Pignel

**Affiliations:** 1 Hyperbaric Medicine Unit, Department of Acute Medicine, University Hospitals of Geneva, 1205 Geneva, Switzerland; pierre.louge@hcuge.ch (P.L.); rodrigue.pignel@hcuge.ch (R.P.); 2 CRC & Division of Clinical-Epidemiology, Department of Health and Community Medicine, University of Geneva & University Hospitals of Geneva, 1205 Geneva, Switzerland; angele.gayet-ageron@hcuge.ch; 3 Emergency Department of Mont-Blanc Hospitals, The Mont-Blanc Hospitals, 74700 Chamonix-Sallanches, France; f.champly@ch-sallanches-chamonix.fr; 4 Hyperbaric Center, Hospices Civils de Lyon, University Hospitals of Lyon-1, 69003 Lyon, France; thierry.joffre@chu-lyon.fr; 5 Division of Medical Information Sciences, Geneva University Hospitals and University of Geneva, 1205 Geneva, Switzerland; christian.lovis@hcuge.ch

**Keywords:** frostbite, classification, hyperbaric oxygen therapy, cold disease, prognosis, amputation, medical outcome

## Abstract

*Background and Objectives*: Frostbite is a freezing injury that can lead to amputation. Current treatments include tissue rewarming followed by thrombolytic or vasodilators. Hyperbaric oxygen (HBO) therapy might decrease the rate of amputation by increasing cellular oxygen availability to the damaged tissues. The SOS-Frostbite study was implemented in a cross-border program among the hyperbaric centers of Geneva, Lyon, and the Mont-Blanc hospitals. The objective was to assess the efficacy of HBO + iloprost among patients with severe frostbite. *Materials and Methods:* We conducted a multicenter prospective single-arm study from 2013 to 2019. All patients received early HBO in addition to standard care with iloprost. Outcomes were compared to a historical cohort in which all patients received iloprost alone between 2000 and 2012. Inclusion criteria were stage 3 or 4 frostbite and initiation of medical care <72 h from frostbite injury. Outcomes were the number of preserved segments and the rate of amputated segments. *Results*: Thirty patients from the historical cohort were eligible and satisfied the inclusion criteria, and 28 patients were prospectively included. The number of preserved segments per patient was significantly higher in the prospective cohort (mean 13 ± SD, 10) compared to the historical group (6 ± 5, *p* = 0.006); the odds ratio was significantly higher by 45-fold (95%CI: 6-335, *p* < 0.001) in the prospective cohort compared to the historical cohort after adjustment for age and delay between signs of freezing and treatment start. *Conclusions*: This study demonstrates that the combination of HBO and iloprost was associated with higher benefit in patients with severe frostbite. The number of preserved segments was two-fold higher in the prospective cohort compared to the historical group (mean of 13 preserved segments vs. 6), and the reduction of amputation was greater in patients treated by HBO + iloprost compared with the iloprost only.

## 1. Introduction

Frostbite is an injury caused by freezing of the skin and underlying tissues. Severe frostbite is a relatively uncommon event that can lead to early arthritis, tissue loss, or amputation. Frostbite comprises on average 2% of mountain emergencies in the western Alps [1]. Frostbite takes place in three phases: pre-freeze/freezing, thawing/rewarming, and mummification.

**Figure 1:**
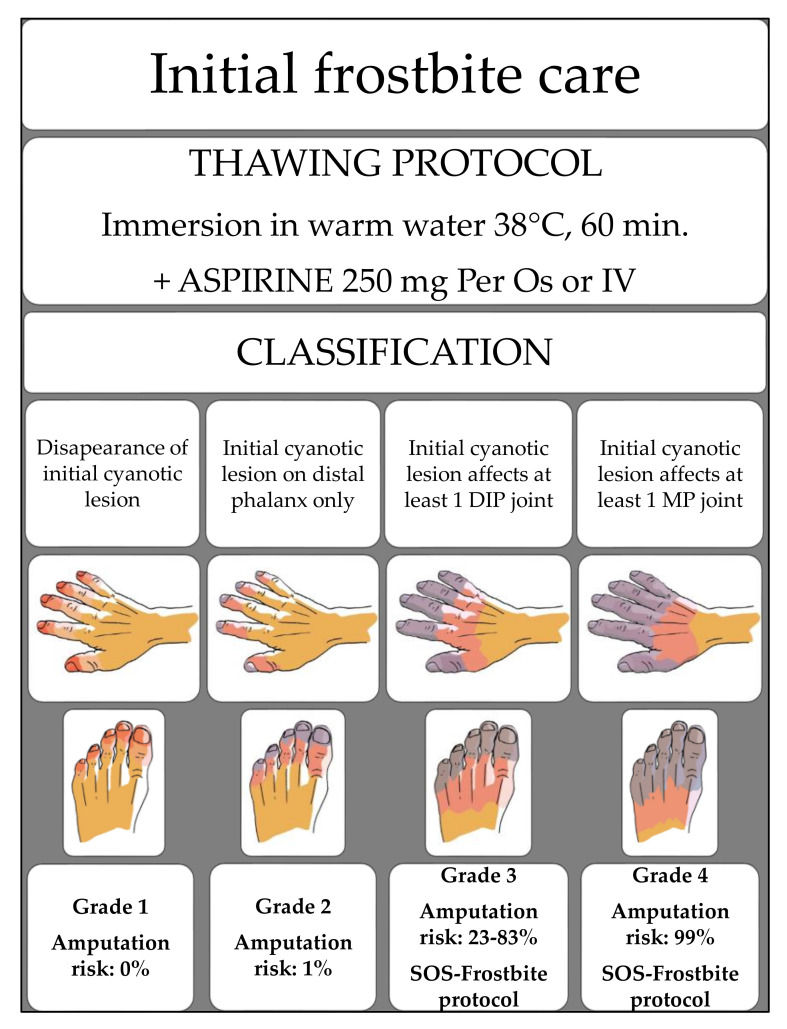
The frostbite classification by E. Cauchy (*drawings@copyright ifremmont*).

Pre/freeze is an acute ischemia with peripheral vasoconstriction. During freezing, cell death is triggered by intracellular dehydration and direct damage to cell membrane by ice microcrystals. Thawing is best accomplished by the immersion of frozen limbs in warm water. After blood flow is restored, cyanotic lesions can occur. During rewarming, there is a vascular stasis with a prothrombotic environment (hypoxia and acidosis), interstitial edema, and ischemia–reperfusion injuries. It leads to the destruction of microcirculation and cell death [2,3]. Frostbite outcome is related to the initial cyanotic lesion. The Cauchy classification defines four grades that predict the amputation risk after rapid thawing in warm water when there is no targeted frostbite care [3]. It is based on the extent of the initial cyanotic lesion. Frostbite is classified as grade 1 if cyanosis disappears, grade 2 if only distal phalanges are cyanotic (amputation risk below 1%), grade 3 if cyanosis involves the intermediate or proximal phalanges (amputation risk: 30–83% greater in the hands than feet), and grade 4 if cyanosis involves the metacarpals or metatarsals (amputation risk: 99%) [3] (Figure 1).

The goal of treatment is to limit tissue damage from hypoxia and acidosis, mitigate the subsequent prothrombotic cascade, reduce edema and the inflammatory response, and minimize the impact of the ischemic–reperfusion syndrome. Prior studies have demonstrated the efficacy of thrombolytics such as recombinant tissue plasminogen activator (rt-PA) [4] and vasodilator such as iloprost [5,6,7] in improving outcome [8]; medical care must be initiated within 24 h for rt-PA [9,10] and 48 h for iloprost [11]. Currently, the Wilderness Medical Society guidelines do not recommend HBO treatment for frostbite [8]. However, HBO may improve frostbite outcome by increasing the cellular oxygen availability to the damaged tissues. This may help to mitigate the negative impact of the inflammatory cascade and the ischaemia–reperfusion syndrome [12]^.^ Few case reports suggest that HBO might improve frostbite injury outcome [13,14,15,16,17,18,19,20,21,22,23]. There are no randomized controlled trials (RCT) with HBO conducted so far. It is arduous to carry out a double-blinded RCT for HBO because frostbite is uncommon, and blinding subjects to HBO or not HBO could be difficult.

We implemented a cross-border European program (INTERREG-IV FRANCE-SUISSE) to foster and coordinate the care management of patients who suffer frostbite in the French and Swiss Alps.

## 2. Materials and Methods

### 2.1. Study Oversight

The SOS-Frostbite research program was a multicenter prospective, non-randomized study from 2013 to 2019. The study was conducted by the hyperbaric centers of Geneva and Lyon, and the Mont-Blanc hospitals in Chamonix and Sallanches. The statistical analysis was performed independently by the unit of methodological support from the CTU of Geneva University Hospital. The study aim was to assess whether the early addition of HBO to standard care with iloprost (prospective group) was associated with better frostbite outcomes compared to standard care alone (retrospective group).

### 2.2. Setting and Participants

Patients were eligible for the SOS-Frostbite protocol after screening determined no contraindication to aspirin, iloprost, or HBO. The inclusion criteria for both groups were grade 3 or 4 frostbite according to the Cauchy classification [3] and start of medical care within 72 h from frostbite injury, which was defined previously in the historical cohort as the onset of frostbite. Physicians involved in the study systematically searched for the onset of loss of sensitivity in the fingers or toes through the medical history to determine this time period.

To identify the historical cohort, we retrospectively collected data of all frostbite medical files treated at the Mont-Blanc hospital from 2000 to 2012. Before 2000, as the Cauchy classification had not yet been established, no patients could be included. All eligible patients who met the inclusion criteria from the retrospective analysis were included in the historical cohort. They were all grade 3 or 4 frostbitten patients who received a standardized protocol including iloprost, which was initiated no longer than 72 h from frostbite injury.

The standardized frostbite treatment: frostbitten extremities were rewarmed by immersion in warm water (38 °C) for 60 min, and patients were given aspirin 250 mg orally. During the hour following the rewarming, the frostbite classification was determined. Grade 3 or 4 frostbite patients received the first iloprost infusion immediately (by infusion pump, 8–10 mcg/h for 6 h, 48–60 mcg/day). Patients were hospitalized for 7 days to continue daily iloprost (by infusion pump, 8 to 10 mcg/h for 6 h, 48–60 mcg/day), aspirin (250 mg/day; orally), antibiotics (amoxicillin/clavulanate: 1 g/125 mg 3 times daily, orally for 7 days), and daily wound care with topical hyaluronic acid.

To identify the SOS-Frostbite group, data were prospectively collected from patients satisfying inclusion criteria who received the same standardized frostbite treatment protocol plus early HBO from 2013 to 2019.

**Figure A1:**
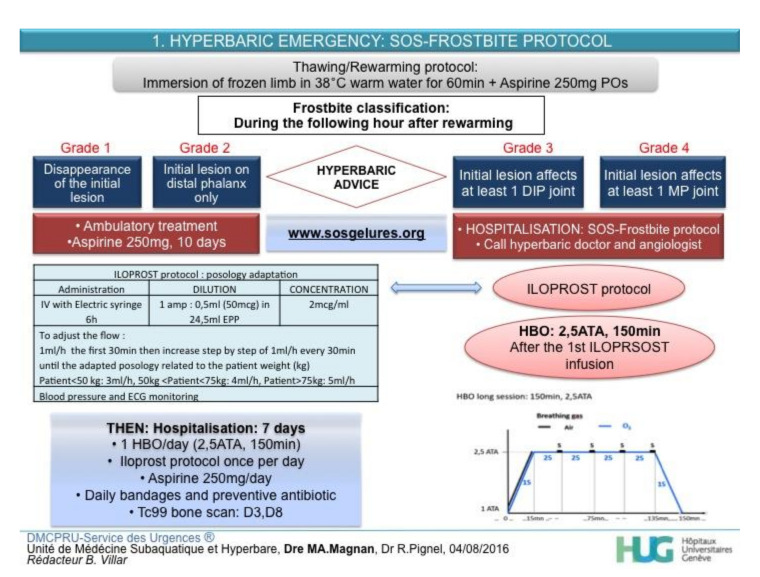
The SOS-Frostbite protocol during hospitalization at university hospitals of Geneva.

The SOS Frostbite protocol: The SOS-Frostbite protocol was initiated upon hospital arrival. Patients were treated with the same standardized protocol as the historical cohort with the addition of HBO. The first HBO (150 min at 2.5ATA) session was done as soon as possible after the first iloprost infusion (from 1 to 6 h after the end of the iloprost infusion, as some patients were transferred from other hospitals to the Geneva or Lyon hyperbaric chamber for HBO). Then, patients were hospitalized for 7 days and received the same treatment protocol as in the historical cohort plus HBO sessions (150 min, 2.5 ATA, 1 daily) (Appendix A, Figure A1). After hospital discharge, the patient completed daily HBO sessions for 7 additional days (14 HBO session in total). Hyperbaric chambers involved in the study used multiplace chambers and patients breathed oxygen via a mask or a hood.

### 2.3. The Follow-Up

A Technecium 99 (Tc99) bone scan was performed at day 3 and day 7 (control group and prospective cohort). Results were considered pathological when the bone scan demonstrated absent or markedly decreased uptake of the Tc99 tracer in the bone tissue (severe bone ischemia). An additional Tc 99 bone scan was conducted at the end of the HBO sessions if radiological improvement (recovery of bone activity) was identified on the day 7 Tc99 bone scan compared to the day 3 Tc99 bone scan. All patients had a clinical examination at 6 months, 1 year. Patients enrolled in the first 4 years of the study also had a follow-up at 2 years and 3 years to evaluate early and delayed sequelae such as arthritis.

### 2.4. Outcomes

The study’s primary outcome was the number of preserved segments at 12 months, which was defined as the difference between the number of segments with frostbite after rewarming and lost segments. Each phalanx and each metacarpal or metatarsal is defined as a segment; 4 segments comprise a ray (3 segments for the thumb or the hallux), and 3 out 4 segments make a digit (2 out 3 segments make the digit for the thumb or the hallux). To align with the eligibility criteria regarding frostbite severity (grade 3 or 4), we only considered rays with at least 2 segments damaged. The secondary outcomes were the number of amputated segments at 12 months and the ratio of the number of amputated segments at 12 months divided by the number of segments with initial frostbite injury.

### 2.5. Data Collection

All data from the prospective and the historical cohorts were collected on site using a standardized case report form. All observations were coded to preserve patient anonymity and data confidentiality.

### 2.6. Statistical Analysis

There was no preliminary estimation of study sample size; we used all available data on 31 December 2019 and obtained a fixed sample size of 58 patients. In the control group, we described 6 (mean ± SD, 5.3) preserved segments at 12 months post-treatment. We had 80% power to detect a two-fold increase in the number of preserved segments (+6) in the standard care plus HBO group, considering a larger variability of the difference of number of preserved segments (±10).

Continuous variables were reported as mean ± SD, median, and interquartile range. Categorical variables are reported as frequencies and percentages. We compared two cohorts of patients: those included between 2000 and 2012 (historical cohort) and those included after 2013 (prospective cohort). We compared continuous variables between the two cohorts of patients with the use of nonparametric Mann–Whitney test, as we anticipated that continuous variables are non-normally distributed and do not respect the assumptions for using Student’s *t*-test; we compared categorical variables between the two cohorts with the use of chi-square or Fisher’s exact tests, depending on assumptions, and *p*-values of less than 0.05 were considered to indicate statistical significance. Since the main outcome (number of preserved segments) was an ordinal variable (0, 1, 2, 3, and 4 preserved segments) and because one patient could have several data points for the main outcome (repeated measurements), we performed mixed ordinal logistic regressions with the patient identifier as a random factor. We compared the main outcome between the two cohorts of patients (HBO plus standard care vs. standard care alone). We adjusted the analysis for patient age, delay between signs of freezing and medical treatment received (<6 h, 6–12 h, 12–24 h, 24–48 h, and 48–72 h). For secondary outcome, we also performed mixed ordinal logistic regressions models as the number of amputations was also ordinal (3–4, 2, 1, 0 amputation), and we also adjusted the analysis for patient age and the delay between signs of freezing and medical treatment received. All analyses were performed with the use of STATA 16 IC (StatCorp, College Station, TX, USA).

## 3. Results

### 3.1. Description

#### 3.1.1. Patients

The prospective cohort: Thirty-nine patients with grade 3 or 4 frostbite were treated from 2013 to 2019 with the SOS-Frostbite protocol; 11 patients were excluded because medical care delay was over 72 h from frostbite injury or the treatment protocol was interrupted or changed. For statistical analysis, 28 patients were prospectively included in the SOS-Frostbite group. None of the patients from the prospective cohort suffered from HBO side effects.

**Figure 2:**
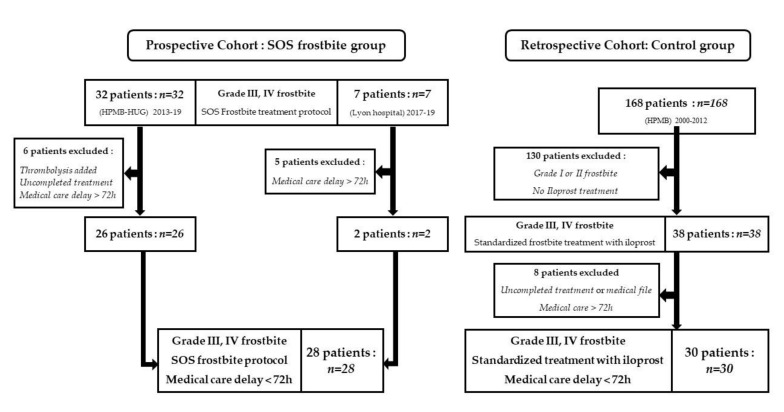
Study flow chart.

The retrospective cohort (control group): After reviewing all frostbite medical files in the Mont-Blanc hospitals (168 medical files), 30 patients met the inclusion criteria (standardized frostbite treatment with iloprost, grade 3 or 4 frostbite and medical care initiated within 72 h from frostbite injury) (Figure 2).

The SOS-Frostbite group and the historical control group both consisted of a similar number of patients with identical inclusion criteria.

The comparison of patient characteristics is presented in Table 1. Percentages of patients with delays of 12 to 24 h or 24 to 48 h were more frequent in the prospective cohort compared to the historical cohort. Patients were significantly older in the prospective than in the historical cohort. A higher proportion of patients with three or four segments with frostbite were observed in the prospective cohort compared to the control group (*p* < 0.001).

#### 3.1.2. Outcomes

A significantly higher mean number of preserved segments per patient was observed in the prospective SOS-Frostbite group (13 SD ± 10) compared to the historical control group (6 SD ± 5) (*p* = 0.006). In the prospective cohort, 57% of patients had three to four preserved segments (respectively 43% for three segments and 14% for four segments) compared to 13% in the control group (respectively 13% for three segments and 0% for four segments). (*p* < 0.001, Table 2). At baseline, a higher but not statistically significant number of frostbitten segments was observed in the prospective than in the control group. However, a significantly higher number of frostbitten amputated segments was observed in the control than in the prospective group (*p* = 0.014, Table 2).

The odds ratio of the number of preserved segments was significantly higher by 20-fold (95%CI: 4-101, *p* < 0.001) in the prospective group who received standard care plus HBO compared to the control group (Table 3, model 1). This association remained after adjustment for patient age and delay between signs of freezing and medical treatment start (Table 3, model 2).

The association between the treatment received (cohort group) and a lower number of amputated segments was assessed. The odds of fewer amputated segments were significantly higher in the prospective group with standard care plus HBO compared to the control group with standard care alone (odds ratio 0.015; 95% CI: 0.0009; 0.25, *p* = 0.003). This association was reinforced after adjustment for patient age and delay between signs of freezing and onset of medical treatment, but due to very small numbers, the imprecision of the estimates was very large (odds ratio 0.0004; 95% CI: 0.00003; 0.06, *p* = 0.002).

If we consider the ratio of segment amputation to all injured segments, a higher proportion of patients with one-third, half, two-thirds, or the total of segments amputated in the control group were observed compared to the standard care plus HBO group after 1-year follow-up (Table 2).

## 4. Discussion

This observational study is the first published prospective study reporting data on severe frostbite treated by early HBO.

In this study, HBO is a positive adjunct to treatment with iloprost. When started within 48 h from injury, iloprost can increase the segment salvage rate up to 78% in severe frostbite [24]. Iloprost has the highest recommendation level in frostbite treatment [8] and should be considered on grade 3 or 4 frostbites when rt-PA is contraindicated or is used in the field. Frostbite treatment with iloprost is strongly recommended, as it decreases the risk of amputation; HBO further improves segment salvage even if initiated after 48 h from frostbite injury.

This study did not compare the combined effect of thrombolytics and HBO. Thrombolytics are another recommended treatment that can lower the amputation rate from 41% to 10% when done within 24 h from frostbite injury [4]; a risk–benefit analysis should always be performed regarding bleeding risk and all contraindication to the treatment.

HBO is a non-invasive treatment; side effects are self-limiting and can mostly be avoided with appropriate screening [25]. In appropriate indications, the benefits of HBO frequently outweigh the risks. The US Food and Drug administration approved HBO for the treatment of acute ischemia, whereas iloprost has not yet been approved for such treatment. It can be performed on some people with contraindication to rt-PA due to the bleeding risk or in children. When available, HBO may be considered as an alternative treatment when there are contraindications to iloprost or thrombolytics. In our study, we showed that HBO plus standard care including iloprost significantly reduced the amputation risk even over 48h from frostbite injury.

The physiological mechanism of HBO action is well known [12,13,14,15,16,17,18,19,20,21,22,23], but there are no previous randomized controlled trials conducted to evaluate the added value of HBO on frostbite injury outcomes. Regarding frostbite physiopathology, there are good reasons as to why HBO could improve frostbite injury outcomes. HBO has a direct action on tissue ischemia, increasing dissolved oxygen and improving oxygen transportation in the blood. The HBO decreases blood viscosity and minimizes the inflammatory cascade. There is a hyperoxic vasoconstriction in the micro vascularization of healthy tissues, inducing a redistribution of blood to hypoxic territories. Those effects of HBO on vasoconstriction decrease edema and the incidence of compartment syndrome. There is a reduction of the deleterious influences of ischemia–reperfusion [12,26,27] besides diminishing damages due to the thaw–rewarming phase; HBO has an anti-infective activity due to its bactericidal effect on anaerobic germs and bacteriostatic action on aerobic germs so it can prevent infection during the mummification phase [12,28]. Finally, when repeated every day, HBO sessions induce vascular endothelium growth factor activation, fibroblast and collagen production, and thus the progression toward the resolution of tissue damage. HBO promotes the formation of the healing sulcus between necrotic and healthy tissues [12,28]. These clinical effects were described in recent retrospective studies [13].

Regarding the longer delay for medical care in the prospective cohort, the second aim of this INTERREG project was to set up a network for severe frostbite management. A SOS-Frostbite call center has been created. Some patients have been repatriated from far away to benefit from this research protocol, which could explain the longer delay for medical care from frostbite injury in the SOS-Frostbite group. Despite the longer delay for medical care in the SOS-Frostbite group, segment salvage was still significantly improved.

## 5. Conclusions

The SOS-Frostbite program is the first controlled prospective study that evaluates the effect of early HBO additive to iloprost on severe frostbite. Results show more favorable outcome in terms of the functionality and quality of life in patients treated by HBO: HBO added to the standard care with iloprost might improve frostbite injury outcomes by doubling the chance to preserve the number of injured segments from amputation.

Moreover, the benefits of HBO frequently outweigh the risks as contraindications and side effects are limited, in comparison to standard treatments such as rt-PA and iloprost. Transferring the patient suffering from severe frostbite to a hyperbaric center could be considered even if it implies delayed HBO, as it still improves frostbite outcomes after 48 h. Our findings should be tested in a randomized controlled trial before concluding that HBO should be added to standard care of severe frostbite in patients receiving iloprost.

## 6. Patents

The decision to design a prospective single arm study instead of two-arm randomized study was made because severe frostbite is an infrequent event [1,2]. We collected data on a small sample of 28 patients prospectively and compared the prospective cohort with data from a retrospective cohort from a previous double blinded RCT [5]. In both series, patients were mostly healthy, had little comorbidity, and had good access to medical care. Frostbite also occurs secondary to occupational exposure and in the homeless and migrant populations. The prognosis and outcome of frostbite for members of socially disadvantaged groups is likely much more severe. The fact our patients were healthy was an advantage, as frostbite was the only injury studied, inducing less bias from other pathologies. The Lyon hyperbaric site was more focused on the treatment of occupational accidents and injuries sustained by homeless patients. These patients were often hospitalized on medical services to treat comorbid conditions with an unfortunate delay in frostbite treatment. These patients were excluded if frostbite treatment was not initiated with 72 h.

Our study was not a randomized controlled trial. We tried to minimize selection and information biases using strict eligibility criteria. The allocation of the treatment group was not at random in our study, but we prespecified a list of criteria to select patients with very similar characteristics in this observational study in order to allow an unbiased comparison of treatment effects between the two treatment groups.

The two groups have a comparable number of patients, but those from the prospective group were older, had more severe frostbite, and the medical care delay was longer in comparison with the control group.

Another hypothesis is that HBO might prevent other side effects such as early arthritis by augmenting the healing process. It is still too early to present data, and it will not be possible to compare data with the historical cohort as there was no long-term follow up over 12 months.

## Figures and Tables

**Table 1:** Description of patients included in the study (*n* = study), the number of preserved digits, and the number of amputated segments.

Variables	Overall	Control Group	SOS Frostbite Group	*p*
		(*n* = 30)	(*n* = 28)	
Age at enrollment, mean ± SD (median: interquartile range), years	33 ± 11 (31: 26–40)	30 ± 9 (27: 25–35)	37 ± 12 (32: 28–43)	0.024 *
Sex, *n* (%)				0.344 **
Male	54 (93)	29 (97)	25 (89)	
Female	4 (7)	1 (3)	3 (11)	
Delay between frostbite and treatment, *n* (%)				<0.001 **
<6 h	6 (10)	5 (17)	1 (4)	
6–12 h	13 (22)	12 (40)	1 (4)	
12–24 h	19 (33)	10 (33)	9 (32)	
24–48 h	18 (31)	3 (10)	15 (54)	
48–72 h	2 (4)	0 (0)	2 (7)	
Frostbite location, *n* (%)				0.424 ***
Right hand	21 (18)	10 (15)	11 (22)	
Left hand	25 (22)	12 (18)	13 (27)	
Right foot	36 (32)	22 (34)	14 (29)	
Left foot	32 (28)	21 (33)	11 (22)	
Number of segments with frostbite, *n* (%)				<0.001 **
2	128 (54)	72 (67)	56 (43)	
3	89 (37)	32 (30)	57 (43)	
4	21 (9)	3 (3)	18 (14)	

* Mann–Whitney nonparametric test; ** Fisher’s exact test; *** Chi-square test.

**Table 2:** Comparison of outcomes between retrospective and prospective cohort studies.

Variables	Overall	Control Group (*n* = 30)	SOS Frostbite Group	*p*
			(*n* = 28)	
Number of preserved segments per patient, mean ± SD (median: interquartile range)	9 ± 9 (6: 3–14)	6 ± 5 (4: 2–9)	13 ± 10 (8: 4–22.5)	0.006 *
Number of segments preserved, *n* (%)				<0.001 **
0	17 (7)	17 (16)	0 (0)	
1	12 (5)	10 (9)	2 (1)	
2	121 (51)	66 (62)	55 (42)	
3	70 (29)	14 (13)	56 (43)	
4	18 (8)	0 (0)	18 (14)	
Total number of rays among frostbite at baseline (*n* = 387), *n* (%)	21 (5)	3 (1)	18 (10)	0.124 ***
Total number of rays amputated among rays with frostbite at baseline (*n* = 21), *n* (%)	2 (10)	2 (40)	0 (0)	0.014 ****
Amputations per patient ±SD	1 ± 4 (0: 0–0)	2 ± 6 (0: 0–1)	0.1 ± 0.3 (0: 0–0)	0.044 *
(median: interquartile range)				
Ratio of amputation/injured digits				<0.001 ****
nil	353 (92)	179 (85)	174 (98)	
One-third	4 (1)	3 (1)	1 (1)	
One-half	8 (2)	6 (3)	2 (1)	
Two-thirds	5 (1)	5 (24)	0 (0)	
1	17 (4)	17 (8)	0 (0)	

* Mann–Whitney nonparametric test. ** Mixed ordinal logistic regression model with number of preserved digits coded as 0, 1, 2, 3, and 4 (five categories) as the dependent variable and group as the independent variable. *** Mixed logistic regression model with beam with frostbite (yes/no) as the dependent variable and group as the independent variable among observations with at least one segment with frostbite. **** Fisher’s exact test.

**Table 3:** Association between treatment group and study outcome, univariate and multivariate analyses.

Number of Preserved Digits (Primary Outcome)	Odds Ratio	95%CI	*p*-Value
Model 1 (univariate analysis)			
Treatment received			<0.001
Standard	1	-	
Standard + HBOT	20	4–101	
Model 2 (multivariable analyses)			
Treatment received			<0.001
Standard	1	-	
Standard + HBOT	45	6–335	
Delay between signs of freezing and medical treatment			0.406
<6 h	1	-	-
6–12 h	2	0.08–40	0.702
12–24 h	1	0.06–21	0.951
24–48 h	0.3	0.01–6	0.389
48–72 h	3	0.03–259	0.659
Age of patient at enrollment	1	0.93–1	0.941

## Data Availability

All data from the prospective and the historical cohorts were collected on site using a standardized case report form in the international frostbite registry. All observations were coded to preserve patient anonymity and data confidentiality.
